# Association between sanitary toilet coverage rate and intestinal infectious disease in Jiangsu Province, China

**DOI:** 10.1038/s41598-021-92291-z

**Published:** 2021-06-17

**Authors:** TingTing Chen, Kraiwuth Kallawicha

**Affiliations:** College of Public Health Sciences, Chulalongkorn University, 11th floor (Room no. 1110) Institute Building 3, Soi Chulalongkorn 62, Phyathai Rd, Pathumwan, Bangkok, 10330 Thailand

**Keywords:** Diarrhoea, Disease prevention, Public health

## Abstract

Intestinal infectious disease is one of the most common diseases in China and is prevalent worldwide. The Chinese government launched a toilet improvement campaign to improve sanitation and reduce the incidence of diseases. This study determined the association between sanitary toilet use and intestinal infectious disease incidence in Jiangsu Province, China during 2011–2019. This study adopted an ecological retrospective research design. All secondary data were obtained through government websites and government information disclosure channels. Multiple linear regression was employed to analyze the association between the incidence of intestinal infectious diseases and sanitary toilet coverage rate and other potential predictors. Data suggested that the aggregate annual incidence of Type A and B intestinal infectious diseases showed a downward trend, the aggregate annual incidence of other infectious diarrhea continued to increase, and hand–foot–mouth disease occurred every other year with the highest annual incidence rate. The incidence was higher in coastal cities. Multiple regression results indicated that the usage of three types of sanitary toilets, compliance rate of water quality, and average ambient temperature have an impact on intestinal infectious diseases. The aggregate annual incidence of Type A and B intestinal infectious disease was negatively correlated with the cumulative use of sanitary toilets (*β* = − 0.036) and surface water quality (*β* =  − 0.135; *p* < 0.05). Increase in sanitary toilet use and water quality control can reduce the number of new cases, which will be beneficial for the population in the province. Moreover, the toilet improvement interventions should continue to maintain high-quality construction.

## Introduction

Intestinal infectious diseases are one of the most common diseases prevalent worldwide. These diseases place a significant health burden on individuals and communities at large, and the morbidity resulting from these diseases are high even in developed countries^1^. A study indicated that intestinal infectious diseases affect approximately 25% of the UK population annually, resulting in a damage of approximately 1.5 billion pounds to the economy, population, and the National Health Service each year^2^. Furthermore, intestinal infectious diseases are the leading cause of death in South Asian countries^3^. Many studies have reported the cause of these diseases including consumption of microbe-contaminated food, lack of clean water, or high levels of poverty^4–6^. One of the crucial problems that lead to high prevalence of these diseases is the lack of sanitary toilets and poor hygienic conditions^7^. These impediments to public health have been observed worldwide, particularly in rural areas.

Because of inadequate toilet sanitation, human feces are directly exposed to air, in turn providing a favorable environment for the breeding of mosquitoes and flies. In addition, because of improper cleaning of feces, many parasites and bacteria present in the feces spread through soil and water sources and become the main source of various diseases, particularly intestinal infectious diseases. Some studies have highlighted that poor toilet hygiene is a risk factor for intestinal infectious diseases; however, the association between sanitary toilets and intestinal infectious diseases has not been supported by any direct evidence^8,9^. The World Bank estimates that inadequate sanitation and unsafe discharge of toilet water results in approximately 1.5 million children getting infected with intestinal infections each year^10^. Furthermore, many studies in developing countries have reported that limited access to toilets can increase intestinal infectious disease incidence with children being the most vulnerable group^11–13^.

China is among the countries affected by high intestinal infectious disease prevalence, and its incidence is especially conspicuous in rural areas^14^. The practice of collecting human waste for the purpose of agricultural fertilization since ancient times has been the source of toilet hygiene problems in China. The prevention and control of diseases did not have a smooth implementation, resulting in the incidence of intestinal infectious diseases being markedly high. This health crisis has received more attention after the founding of the People’s Republic of China^15^. In 1950s, because of the poor conditions of urban and rural environments and the threat of germ warfare in China, the Patriotic Health Campaign was launched nationwide, from China’s toilet improvement initiative originated^16^. The initiative gradually gained momentum through numerous national campaigns, and the pilot construction of rural sanitary toilets began occurring throughout the country. In 2015, the government began paying more attention to toilet improvement, as per the instruction of the General Secretary, Xi Jinping, and the campaign was extended to vast rural areas^17^. However, approximately 17 million households in China still face serious sanitation problems because of poor toilet conditions. Previous evidence has suggested that only 48.6% of rural households have access to sanitary toilets, while 2% still do not have toilets^18^. This problem of limited accessibility to toilets can lead to the spreading of intestinal infectious diseases.

Jiangsu Province has been actively implementing the national health work policy, and it ranks first in terms of health promotion. The rural toilet improvement in Jiangsu Province has been at the forefront of the country’s toilet improvement initiative, leading to the popularity of sanitary toilets growing rapidly in the province^16^. However, the latest data released in 2019 show that intestinal infectious diseases are still among the common infectious diseases affecting Jiangsu Province, accounting for approximately 45% of all mandatory reported infectious diseases^19^.

To our knowledge, limited attention has been paid to the improvement of toilets and intestinal infectious diseases in Jiangsu Province. Therefore, in this study, we investigated the association between the increase in sanitary toilets and the incidence of intestinal infectious diseases in Jiangsu Province of China during 2011–2019. In addition, water resource quality, vector density, population density and other environmental factors were considered in the analysis.

## Materials and methods

### Study design

This study employed an ecological retrospective design to study the association between the increase of sanitary toilets and intestinal infectious diseases between 2011 and 2019 in Jiangsu Province. This province is located on the eastern coast of China with a total population of 80.7 million as of 2019. The analysis of this study was conducted at two levels. First, Jiangsu Province was considered a single unit. Second, 13 prefecture-level cities (i.e., Nanjing, Wuxi, Xuzhou, Changzhou, Suzhou, Nantong, Lianyungang, Huaian, Yancheng, Yangzhou, Zhenjiang, Taizhou, and Suqian) of Jiangsu Province were analyzed separately as the second level.

### Ethical review

The study protocol followed the Declaration of Helsinki of 1975, as revised in 1983. Secondary data were obtained in the province and city levels. None of individual data were exposed or used in the analysis. Therefore, the informed consent was waived. The study protocol was approved and the informed consent was waived by the Research Ethics Review Committee for Research involving Human Research Participants, Group I, Chulalongkorn University (Ethic No. 102/2020).

### Data collection

#### Dependent variable (intestinal infectious diseases)

Intestinal infectious diseases were analyzed in this study on the basis of mandatory reported infectious diseases in China. Three major types (six diseases) of intestinal infectious diseases were studied: Type A, which included cholera (ICD-10-A00); Type B, which included typhoid (i.e., typhoid [ICD-10-A01.0] and paratyphoid [ICD-10-A01.1-A01.4]), dysentery (i.e., bacillary dysentery [ICD-10-A03.0] and amoebic dysentery [ICD-10-A06.0]), and viral hepatitis (i.e. hepatitis A [ICD-10-B15.], hepatitis E [ICD-10-B17.2], and untyped hepatitis [ICD-10-B19]); and Type C, which included other infectious diarrhea (i.e., infectious diarrhea other than cholera, bacterial and amoebic dysentery, and typhoid fever and paratyphoid fever [ICD-10-A09]) and hand–foot–mouth disease (HFMD) [ICD-10-B08.4]. The characteristics of each infectious disease studied here, including types of pathogens, modes of transmission, and average incidence, are presented in Supplement material Table S1. These data were retrieved from the Jiangsu Provincial Health Commission through official websites and government information disclosure applications.

The aggregate annual incidence of all intestinal infectious diseases (three types) for each year was calculated as:

1 $${\text{Aggregate}}\;{\text{annual}}\;{\text{incidence}} = \frac{{{\text{Total}}\;{\text{no}}.\;{\text{of}}\;{\text{new}}\;{\text{cases}}\;{\text{of}}\;{\text{diseases}}\;{\text{reported}}\;{\text{each}}\;{\text{year}}}}{{{\text{Total}}\;{\text{population}}\;{\text{of}}\;{\text{Jiangsu}}\;{\text{Province}}\;\left( {{\text{at}}\;{\text{risk}}} \right)}} \times 100,000,$$

whereas the annual incidence of each disease was calculated as:

2 $${\text{Annual}}\;{\text{incidence}} = \frac{{{\text{No}}.\;{\text{of}}\;{\text{new}}\;{\text{cases}}\;{\text{of}}\;{\text{each}}\;{\text{disease}}\;{\text{reported}}\;{\text{each}}\;{\text{year}}}}{{{\text{Total}}\;{\text{population}}\;{\text{of}}\;{\text{Jiangsu}}\;{\text{Province}}\;\left( {{\text{at}}\;{\text{risk}}} \right)}} \times 100,000$$

#### Independent variables

In this study, the term sanitary toilet refers to types of toilets, namely, sanitary toilets, harmless sanitary toilets, and sanitary public toilets. Toilets with fences and roofs and an impermeable and airtight septic tank for waste collection were defined as sanitary toilets. These toilets are clean, free of fly maggots, and odorless. Moreover, feces is removed as and when required. Harmless sanitary toilets referred to those that met the basic requirements of sanitary toilets. In addition, these toilets have facilities for the decontamination of feces and are managed according to specification. Finally, public sanitary toilets referred to the ones that are available in public areas for those who do not have toilets available at home. The structure of each type of toilet is provided in Supplement materials Fig. S1. These data were retrieved from the China Statistical Yearbook on Environment. The sanitary toilet coverage rate was calculated as follows:

3 $${\text{Sanitary}}\;{\text{toilet}}\;{\text{coverage}}\;{\text{rate}} = \frac{{{\text{No}}.\;{\text{of}}\;{\text{households}}\;{\text{using}}\;{\text{various}}\;{\text{types}}\;{\text{of}}\;{\text{sanitary}}\;{\text{toilets}}}}{{{\text{Total}}\;{\text{no}}.\;{\text{of}}\;{\text{rural}}\;{\text{households}}}} \times 100$$

Information on surface water quality was obtained from the cross-sectional data from the national surface water environmental quality assessment conducted by the Water Resources Bureau. The average annual water quality that met the Class III standard (i.e., water that qualified for further processing as drinking water) was included for the analysis. The compliance rate of water quality was calculated as follows:

4 $${\text{Compliance}}\;{\text{rate}} = \frac{{{\text{No}}.\;{\text{of}}\;{\text{qualified}}\;{\text{samples}}}}{{{\text{Total}}\;{\text{no}}.\;{\text{of}}\;{\text{samples}}}} \times 100\%$$

Vector density (i.e., mice, flies, cockroaches, and mosquitoes) was also collected at select monitoring points according to different geographic locations. Data were retrieved from the Department of Ecology and Environment of Jiangsu Province. The density was calculated as follows:

5 $${\text{Vector}}\;{\text{density}} = \frac{{{\text{No}}.\;{\text{of}}\;{\text{vectors}}}}{{{\text{No}}.\;{\text{of}}\;{\text{traps}}}}$$

Rainfall (mm) and temperature (℃) comprised other environmental parameters. Temperature data comprised the annual average temperature and maximum and minimum temperature. These data were retrieved from the Jiangsu Statistical Yearbook.

### Statistical analysis

The distribution of the coverage rate of sanitary toilets in Jiangsu Province and each prefecture-level city from 2011 to 2019 (Eq. 3), trend of the incidence of intestinal infectious diseases (Eqs. 1 and 2), and other independent variables (Eqs. 4 and 5) were described using descriptive statistics. The association between the incidence of three types and each intestinal infectious disease, sanitary toilet coverage rate, and other potential predictor variables were prescreened using a simple linear regression analysis. Independent variables that reached a significance level of *p* < 0.2 were considered for further multiple regression analysis. Independent variables with *p* < 0.05 were considered statistically significant in the final multiple regression model. In addition, the city-specific models were analyzed. However, because of the lack of toilet coverage information in each city, only environmental parameters were included in the analysis. All statistical analyses were performed using IBM SPSS Statistics for Windows (version 22; IBM, Armonk, NY, USA). ArcGIS (version 10.6; Esri, Redlands, CA, USA) was used to illustrate the spatial distribution of the disease incidence.

### Ethical approval

The study protocol was approved and the informed consent was waived by the Research Ethics Review Committee for Research involving Human Research Participants, Group I, Chulalongkorn University (Ethic No. 102/2020).

## Results

### Distribution of intestinal infectious diseases in Jiangsu Province

**Figure 1:**
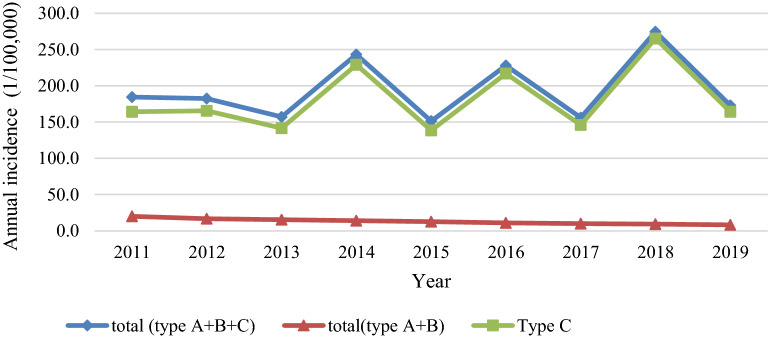
Variation trends in three types of intestinal infectious diseases in Jiangsu Province during 2011–2019.

According to the data from 2011 to 2019 presented in Fig. 1, Type C intestinal infectious disease was the most common type of disease reported to the government, whereas Type A and B intestinal infectious diseases were far less observed. The distribution patterns of Type A and B intestinal infectious diseases showed a clear downward trend, from 20.04 per 100,000 in 2011 to 8.28 per 100,000 in 2019 (Eq. 2), whereas the pattern of Type C intestinal infectious diseases fluctuated with a peak incidence every other year. The distribution pattern clearly indicated that the aggregate annual incidence of three types of diseases (Eq. 1) was generally consistent with that of Type C intestinal infectious diseases.

As demonstrated in Table 1, the annual incidence of Type A intestinal infectious disease was very low. This is because of the very large denominator (total population of Jiangsu Province) compared to the numerator (annual case number) (Eq. 2). Therefore, we combined the annual incidence of Type A and B intestinal infectious diseases for further statistical analysis (Eq. 1). The annual incidence of HFMD was the highest among all studied diseases, accounting for 81% of the total intestinal infectious diseases. Its average incidence in 9 years was 158.2 per 100,000. Other infectious diarrhea had the second highest annual incidence, accounting for 12% of the total intestinal infectious diseases. The annual incidence of viral hepatitis and dysentery accounted for 4% and 3%. The annual incidence of typhoid and paratyphoid and cholera were very low. The distribution of viral hepatitis and dysentery annual incidence decreased over the study period, while the annual incidence of other infectious diarrhea generally increased. Moreover, the fluctuating annual incidence pattern of HFMD, which peaked every other year, showed a similar trend to the aggregate annual incidence of three types of intestinal infectious diseases.

**Table 1:** Annual incidence rate of each intestinal infectious disease in Jiangsu Province for each year during 2011–2019.

Incidence rate of intestinal infectious disease (1/100,000)^a^							
Year	Type A	Type B			Type C		Total
	Cholera	Viral hepatitis	Dysentery	Typhoid, paratyphoid	Other infectious diarrhea	Hand–foot–mouth disease	
2011	0.0	10.3	9.4	0.3	18.3	146.0	184.3
2012	0.0	9.3	7.3	0.3	19.2	146.5	182.5
2013	0.0	9.5	5.7	0.3	20.1	121.7	157.3
2014	0.0	8.8	5.0	0.2	18.7	210.5	243.2
2015	0.0	7.6	4.9	0.2	19.2	119.4	151.3
2016	0.0	6.7	4.1	0.4	19.3	197.6	228.0
2017	0.0	6.4	3.4	0.2	31.9	114.2	156.1
2018	0.0	6.0	3.1	0.2	27.9	237.5	274.6
2019	0.0	5.9	2.8	0.2	33.5	130.7	173.0
Mean	0.0	7.8	5.1	0.3	23.1	158.2	194.5

The permanent residents of Jiangsu Province in 2019 amounted to 80.7 million.

^a^Annual incidence rates were calculated as Eq. (2).

**Figure 2:**
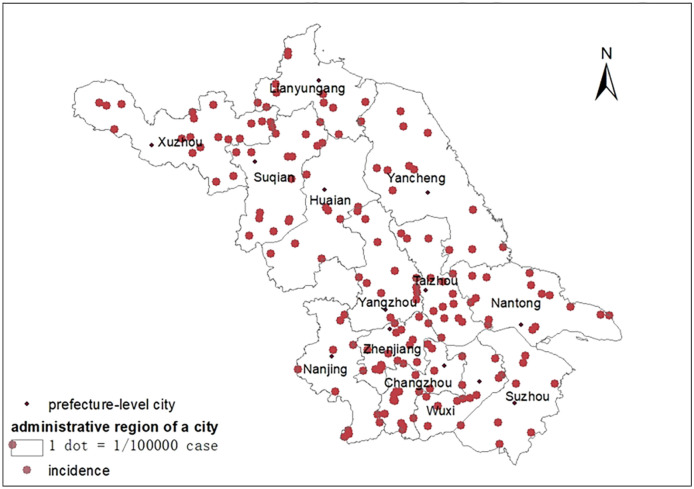
Distribution of the average aggregate annual incidence of Type A and B intestinal infectious diseases in Jiangsu Province during 2011–2019 (Map was created using ArcGIS version 10.6; https://desktop.arcgis.com/en/).

**Figure 3:**
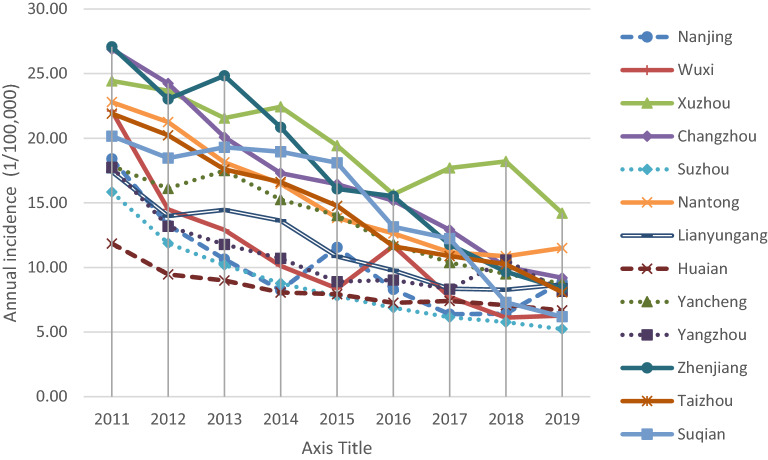
Trends in the average aggregate annual incidence of Type A and B intestinal infectious diseases in 13 cities of Jiangsu Province.

Figures 2 and 3 demonstrate the spatial and temporal distribution of the average aggregate annual incidence of Type A and B intestinal infectious diseases in Jiangsu Province from 2011 to 2019, respectively. In Fig. 2, the dot density map shows the average aggregate annual incidence where one dot refers to 1/100,000 case. In general, the inner cities had lower annual incidence rate compared with the middle and southern cities. The lowest annual incidence was observed in Huai’an city while the highest annual incidence was observed in Xuzhou city. As presented in Fig. 3, the temporal distribution of diseases in each city showed a downward trend from 2011 to 2019, except in Xuzhou city where a reverse pattern was observed since 2016, inconsistent with the patterns in other cities.

### Distribution of toilet coverage rate and environmental parameters

**Figure 4:**
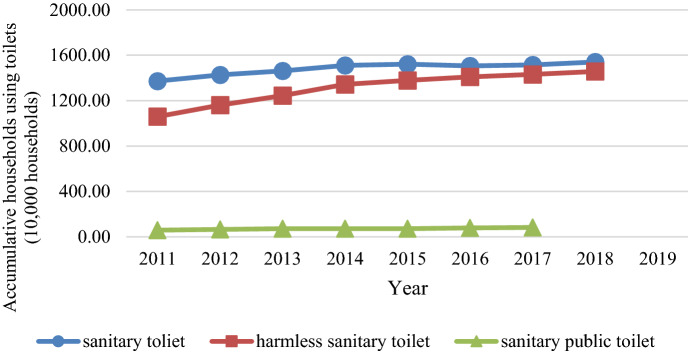
Trends in the cumulative households using three types of sanitary toilets (Unit = 10,000 households).

Figure 4 presents the trends in the cumulative number of three types of toilets from 2011 to 2019 (Eq. 3). The number of each specific type of toilet increased year after year after the government implemented the campaign. The coverage rate of harmless sanitary toilets showed the most change, increasing from approximately 10 million households in 2011 to 15.4 million households in 2019.

Table 2 highlights the vector density and other environmental data analyzed during the study period (Eqs. 4 and 5). Data on the vector density were available from 2011 to 2018. No consistent pattern of change was observed in the overall density of the four pests in Jiangsu Province. The coverage rate of Class III water sources showed an increasing pattern from 36% in 2011 to 78% in 2019, implying that the quality of surface water was consistently improved during the entire study period. Similarly, the average temperature in Jiangsu Province showed a slight increase in the past 9 years, whereas rainfall varied across the study period, reaching 1612.3 mm in 2016.

**Table 2:** Vector density, compliance rate of surface water quality, and average temperature in Jiangsu Province for each year during 2011–2019.

Year	Vector density (Eq. 5)				Surface water (%) (Eq. 4)	Average temperature (°C)	Rainfall (mm)
	Mice	Cockroach	Mosquito	Fly			
2011	0.3	0.7	2.1	3.5	36	15.4	1016.8
2012	0.4	1.0	3.7	3.7	43	15.5	1027.1
2013	0.5	1.0	1.9	4.6	46	16.1	882.4
2014	0.4	1.3	2.3	3.6	46	15.9	1083.3
2015	0.3	1.0	2.5	3.7	48	15.9	1371.1
2016	0.3	0.7	1.4	4.6	68	16.3	1612.3
2017	0.4	0.7	1.9	4.0	71	16.6	1109.6
2018	0.3	0.5	1.8	3.7	68	16.5	1145.7
2019	ND	ND	ND	ND	78	ND	798.5

*ND* no data available.

### Association between intestinal infectious diseases and other variables

Table 3 presents the results of the simple regression analysis of intestinal infectious diseases and potential predictor variables (*p* < 0.2). As expected, the number of households using sanitary toilets and the average temperatures showed a negative association with incidence of diseases. Similarly, the surface water quality and temperature were negatively associated with the incidence of diseases. These variables were further analyzed using multiple regression.

**Table 3:** Association between the aggregate annual incidence of Type A and B intestinal infectious diseases and various factors in Jiangsu Province.

Variables	Type A and B			Viral hepatitis			Dysentery			Typhoid and paratyphoid			Other infectious diarrhea		
	*β*	SE	*p* value	*β*	SE	*p* value	*β*	SE	*p* value	*β*	SE	*p* value	*β*	SE	*p* value
Accumulative households using sanitary toilets	**− 0.060**	**0.009**	**0.001**	**− 0.024**	**0.006**	**0.008**	**− 0.036**	**0.004**	**< 0.001**	**− 0.001**	**0.000**	**0.188**	0.043	0.032	0.228
Accumulative households using harmless sanitary toilets	**− 0.025**	**0.002**	**< 0.001**	**− 0.010**	**0.002**	**0.001**	**− 0.015**	**0.001**	**< 0.001**	0.000	0.000	0.305	**0.021**	**0.012**	**0.131**
Accumulative households using sanitary public toilets	**− 0.429**	**0.056**	**0.001**	**− 0.167**	**0.041**	**0.010**	**− 0.260**	**0.027**	**< 0.001**	− 0.003	0.004	0.550	**0.417**	**0.208**	**0.101**
Density of mice	19.437	28.123	0.515	13.291	11.521	0.293	6.190	16.715	0.724	− 0.074	0.636	0.911	− 15.989	40.059	0.704
Density of cockroach	6.006	5.402	0.309	**3.704**	**2.097**	**0.128**	2.362	3.294	0.500	− 0.063	0.127	0.643	**− 12.165**	**6.575**	**0.114**
Density of mosquito	2.381	1.990	0.277	1.013	0.872	0.290	1.401	1.146	0.267	− 0.033	0.046	0.506	− 2.203	2.942	0.482
Density of fly	− 2.369	3.205	0.488	− 0.798	1.422	0.595	− 1.660	1.814	0.396	0.083	0.065	0.247	0.129	4.650	0.979
Surface water (meets Class III standards and above)	**− 0.239**	**0.033**	**< 0.001**	**− 0.104**	**0.012**	**< 0.001**	**− 0.126**	**0.024**	**0.001**	− 0.001	0.002	0.588	**0.337**	**0.084**	**0.005**
Average temperature	**− 7.778**	**1.351**	**0.001**	**− 3.201**	**0.745**	**0.005**	**− 4.523**	**0.759**	**0.001**	− 0.054	0.072	0.483	9.123	**3.017**	**0.023**
Maximum temperature	− 0.731	1.039	0.508	− 0.267	0.458	0.581	− 0.464	0.596	0.466	− 0.001	0.024	0.983	2.197	**1.204**	**0.118**
Minimum temperature	**− 1.386**	**0.714**	**0.100**	− 0.483	0.345	0.211	**− 0.873**	**0.388**	**0.065**	**− 0.030**	**0.016**	**0.109**	− 0.001	0.001	0.515
Rainfall	− 0.003	0.006	0.676	− 0.002	0.002	0.505	− 0.001	0.003	0.692	0.000	0.000	0.317	− 0.009	0.009	0.337

Bold characters signify Sig at p < 0.2.

In the final multiple regression analysis, two models remained significant (Table 4). The results highlighted that the cumulative number of households using sanitary toilets (*β* =  − 0.036) and the compliance rate of surface water (*β* =  − 0.135) were negatively associated with the aggregate annual incidence of type A and B intestinal infectious diseases in Jiangsu Province (*R*^2^ = 0.996, *p* < 0.05). According to the analysis, the incidence of Type A and B intestinal infectious diseases were aggregated and treated as dependent variable because of the very low annual incidence of Type A. Therefore, the association observed in this part was rather for Type B intestinal infectious disease. Moreover, the results indicated that the cumulative number of sanitary toilets used (*β* =  − 0.016) and sanitary public toilets used (*β* =  − 0.159*)* were negatively associated with the annual incidence of dysentery (*R*^*2*^ = 0.998, *p* < 0.05).

**Table 4:** Multiple linear regression models for intestinal infectious diseases in Jiangsu Province.

Variables	*β*	SE	*p-*value	95% CI	*R*^*2*^
**Type A and B**					
Accumulative households using sanitary toilets	− 0.036	0.003	< 0.001	− 0.043, − 0.029	0.996
Surface water (Meets Class III standards and above)	− 0.135	0.011	< 0.001	− 0.164, − 0.107	
**Dysentery**					
Accumulative households using sanitary toilets	− 0.016	0.002	0.001	− 0.02, − 0.012	0.998
Accumulative households using sanitary public toilets	− 0.159	0.011	< 0.001	− 0.191, − 0.128	

A city-specific multiple regression analysis was performed. Table 5 presents the statistically significant models for only certain cities. Average temperature was the common variable that was significantly associated with the aggregate annual incidence of Type A and B intestinal infectious diseases, and it showed a negative association with the aggregate annual incidence of Type A and B intestinal infectious diseases in Nanjing (*β* =  − 5.091), Nantong (*β* =  − 4.692), Yangzhou (*β* =  − 1.919), and Zhenjiang (*β* =  − 22.644; *p* < 0.05).

**Table 5:** Association between the aggregate annual incidence of Type A and B intestinal infectious diseases and various factors in each city.

Variables	*β*	SE	*p* value	Adjusted *R*^*2*^
**Nanjing City**				
Average temperature	− 5.091	1.756	0.034	0.860
Population density	− 0.023	0.007	0.019	
**Suzhou City**				
Density of mosquito	1.965	0.211	0.001	0.984
Density of fly	− 0.538	0.139	0.018	
Population density	− 0.011	0.001	0.000	
**Nantong City**				
Average temperature	− 4.692	0.705	0.001	0.973
Population density	0.018	0.003	0.003	
**Huaian City**				
Density of mosquito	− 0.675	0.223	0.029	0.742
Population density	0.050	0.016	0.024	
**Yangzhou City**				
Density of mice	11.866	2.312	0.007	0.966
Average temperature	− 1.919	0.462	0.014	
Minimum temperature	− 0.531	0.171	0.036	
**Zhenjiang City**				
Density of fly	6.382	1.468	0.007	0.851
Average temperature	− 22.644	4.259	0.003	
Population density	0.500	0.115	0.007	
**Taizhou City**				
Density of mice	1.533	0.179	0.000	0.934
Density of mosquito	9.268	1.172	0.001	

Only significant models with *p* < 0.05 are shown in the table.

## Discussion

Intestinal infectious diseases remain a health problem in China, particularly in rural areas. The results of the current study highlight the trends of the incidence of various intestinal infectious diseases from 2011 to 2019 in Jiangsu Province and its cities. Although the aggregate annual incidence of Type A and B intestinal infectious diseases was relatively low, their declining trend still warranted observation. This result was comparable with that of the studies conducted in other provinces^20,21^, particularly in Zhejiang Province, the province adjacent to Jiangsu Province^22^.

The annual incidence of Type C intestinal infectious diseases was much higher than that of Type A and B combined together. This can be attributed to the presence of many kinds of pathogens of Type C intestinal infectious diseases and the improvement of diagnostic tools and monitoring network^23^. Another reason is that the government had implemented stricter supervision, reporting, and management for Type A and B intestinal infectious diseases, because it leads more serious health effects than Type C diseases. Epidemiological investigations were conducted in the shortest time to prevent further spreading of the epidemic^24^. The annual incidence of Type C diseases was found to increase year after year, consistent with the results of a study conducted in the Lanzhou city of Gansu Province^25^. Among the different Type C diseases, HFMD disease was the most common one. The annual incidence of this disease was found to peak every other year, similar to the distribution of HFMD reported in studies conducted in other regions of China^26,27^. The annual incidence pattern of Type C diseases observed may be associated with the human immune system. A study indicated the presence of a widespread latent infection in the population. The duration of specific antibodies after getting infected is > 6 months; however, the specific timeframe needs to be further examined^28^. In addition, children aged < 5 years were the most affected by the HFMD. The infant antibody positive rate with 6 months is approximately 30%, and it drops to approximately 10% after 6–12 months. Subsequently, the antibody positive rate can increase by approximately 12% per year and stabilize by the age of 5 ^29^.

During the study period, the distribution of diseases in Jiangsu Province were more clustered in the coastal cities as observed in other regions. The cluster of incidences may be related to the unqualified water quality and the dietary intake of the local residents, such as raw or semi-alive seafood^30^. The internal migration of population to the economically developed areas in the eastern and southern parts of the province could be another reason. However, the cluster was not observed in Suzhou city, the southeastern city of Jiangsu Province. This could be because the economic conditions, urbanization levels, healthcare services, and sanitary conditions of this city is well developed^31^. The high aggregate annual incidence rate of Type A and B intestinal infectious disease in certain cities (i.e., Xuzhou, Suqian, Yancheng, Changzhou, and Nantong) can be attributed to flood disasters. Flood-affected areas experience dramatic changes in environmental conditions, including temperature, rainfall, and humidity. These changes affect the number of pathogens and disease vectors, leading to a higher incidence compared with that in nonaffected areas^32^.

The results of the analysis clearly revealed that increase in sanitary toilet coverage rate led to a decline in the incidence of intestinal infectious diseases in Jiangsu Province. This decline was particularly conspicuous for Type A and B intestinal infectious diseases and dysentery. The popularity of sanitary toilets had clear effects on disease prevention, consistent with the results of another study^33^. A study conducted in Yuxi City, Yunnan Province reported that typhoid and paratyphoid continued to occur frequently because of the inappropriate construction of a flushing and sewage discharge system^34^. Therefore, great attention must be focused on during the construction of toilets, particularly sewer pipes that need to be built according to local conditions. Furthermore, human excreta should be regularly cleaned and protective measures should be taken against water source contamination. These interventions can effectively reduce the density of fly maggots—the dominant vector of intestinal infectious diseases^35^.

This study discovered that toilet improvement was not effective in controlling the most common Type C intestinal infectious diseases. This finding is consistent with that of studies conducted in other provinces and cities in China^36,37^. Therefore, prevention and disease control measures should focus on Type C intestinal infectious diseases through upgradation of toilet improvement technology. Although no positive results have been observed in relevant studies, according to news reports, the incidence of HFMD has been effectively controlled in the Zibo city of Shandong Province and other areas after toilet improvement^38^. Therefore, Enhancing regional communication and sharing experience may be an effective way to improve the effectiveness of toilet improvement.

In addition to the coverage rate of sanitary toilets, the compliance rate of surface water quality was associated with intestinal infectious diseases incidence rate. Surface water is the main source of drinking water. In general, high water quality can lower the risk of intestinal infections. Although people do not drink water from natural sources directly, opportunities for direct and indirect contact with these water sources still exist, such as when water is used for sediment for agricultural purpose, particularly in vegetable fields. The contamination can transfer to human pathogens and result in diseases^39^. Furthermore, water quality was positively associated with other infectious diarrhea. This result was comparable with a study conducted in Suzhou that indicated that the transmission routes of other infectious diarrhea are more complicated (e.g., air droplets and close contact). The existing disinfection technology may not be able to effectively control certain pathogens, leading to higher incidence^37^. Another study identified that drinking habits can also affect the incidence of diseases; that is, residents who drink unboiled water are more likely to get sick compared with those who boil water before drinking^40^. This may explain the association between water quality and other infectious diarrhea incidence.

The impact of meteorological factors on pathogens, hosts, vectors, and susceptible people have been reported. In general, temperature affects the replication and survival of pathogens in the environment, in addition to people’s living habits^41^. This implies that the rise of temperature will increase the incidence of certain diseases. However, this association was not observed in our study at both the province and city levels, as indicated in our final regression models. Although a positive association was anticipated, we found temperature to be negatively associated with Type A and B intestinal infectious diseases. This can be explained by the increasing number of cumulative households using sanitary toilets and improvements in sanitary conditions—both of which play a greater role in controlling diseases. Therefore, the effect of temperature on intestinal infectious diseases was not observed.

While other studies have utilized data from smaller city areas, to our knowledge, our study is a rare analysis that employed data from Jiangsu Province as a whole and each city separately^33,42^. Notably, data on sanitary toilets for 2018 and 2019 in this study were changed on the basis of official information. The current study’s analysis results can better reflect the effect of toilet improvement policy implementation on the incidence of intestinal infectious diseases. However, our study had several limitations. First, the immunization data of vaccine preventable diseases were not available. Hepatitis A vaccine has been included in the immunization program implemented by Jiangsu Province government since 2008, whereas hepatitis E vaccine and E71 vaccine for HFMD were optional^43^. Since immunization reduces incidences of targeted infectious diseases, and since the study did not consider vaccination against three of the infectious diseases (hepatitis A, HFMD caused by enterovirus-71, and hepatitis E), the study was unable to determine the independent contribution of vaccination to the observed decline in intestinal infections. Second, information on toilet coverage and incidence of Type C intestinal infectious diseases in each of the 13 cities were lacking. Our analysis considered only environmental parameters. Therefore, analysis of spatial distribution and the actual association between toilet coverage rate and the incidence rate of intestinal infectious diseases in each city could not be concluded. Third, our study employed an ecological study design to assess the association between toilet coverage rate and annual incidence of diseases. The lack of information on the sample population’s personal characteristics, such as drinking or eating behavior and the toilet-using habits, limited us to perform an analysis at the individual level. Therefore, conclusions and recommendations at the individual level could not be provided. Finally, our study included only the variables that were available (i.e., toilet coverage rate, water quality, and meteorological factors) in the analysis. Various predisposing factors associated with intestinal infectious diseases are still left to be examined. Therefore, further investigation is required, particularly at the individual level.

## Conclusion

The increase in the number of sanitary toilets is significantly related to the decrease in Type A and B intestinal infectious diseases, implying that the popularization of sanitary toilets can directly and effectively prevent and control the incidence of Type A and B intestinal infectious diseases. However, toilet improvement interventions could not effectively control Type C diseases. Considering the coverage rate of sanitary toilets and harmless sanitary toilets has almost achieved full capacity, new technologies could be implemented in the next phase of the toilet improvement campaign. New construction standards can be proposed on the basis of the control of intestinal infectious diseases by current sanitary toilets. Furthermore, particular attention can be paid to later maintenance work. Hygiene promotion should be strengthened and residents should be encouraged to take the initiative to participate in toilet improvement programs that could benefit intestinal infectious disease prevention and control.

## Supplementary Information

Supplementary Information.
