# Supplementary material for: Association between sanitary toilet coverage rate and intestinal infectious disease in Jiangsu Province, China
Source: Sci Rep. 2021 Jun 17;11:12805. doi: 10.1038/s41598-021-92291-z (PMC8211806; doi:10.1038/s41598-021-92291-z)
Supplement: Supplementary file 1 — Supplementary Information. [file 41598_2021_92291_MOESM1_ESM.docx]

**Supplemental materials**

**Table S1** The characteristics of intestinal infectious diseases in this study.

| **Diseases** | **Pathogen** | **Major modes of transmission** | **Average incidence (1/100,000)** |
| --- | --- | --- | --- |
| Cholera | *Vibrio cholerae* O1  *Vibrio cholerae* O139 | Digestive tract transmission (water transmission) | 0.0 |
| Typhoid | *Salmonella typhi* | Digestive tract transmission (water and food) | 0.3 |
| Paratyphoid | *Salmonella paratyphi* |  |  |
| Bacillary dysentery | *Shigella（Sh.flexneri, Sh.sonnei)* | Contact transmission  Digestive tract transmission  Insect-borne transmission | 5.1 |
| Amoebic dysentery | *Entamoeba histolytica* |  |  |
| Hepatitis A | Hepatitis A virus (HAV) | Digestive tract transmission (aquatic shellfish)  Contact transmission | 7.8 |
| Hepatitis E | Hepatitis E virus (HEV) | Digestive tract transmission (water, food)  Contact transmission  Blood transmission (intravenous transfusion)  Mother-to-child transmission (MTCT) |  |
| Untyped hepatitis | Viral hepatitis caused by a virus infection that is not completely clear |  |  |
| Other infectious diarrhea | Bacteria, viruses, fungi, protozoa, etc | Digestive tract transmission  contact transmission  Respiratory transmission | 23.1 |
| Hand–foot–mouth disease | Coxsackie virus A16 (CoxA16)  Enterovirus 71 (EV71) | Respiratory transmission (droplets)  Contact transmission (secretions and blister fluid)  Digestive tract transmission | 158.2 |
| These Intestinal infectious diseases are common diseases transmitted by fecal- oral transmission | | | |

**Figure S1** Structure of sanitary toilet
